# Cost and Outcome of Behavioural Activation versus Cognitive Behavioural Therapy for Depression (COBRA): a randomised, controlled, non-inferiority trial

**DOI:** 10.1016/S0140-6736(16)31140-0

**Published:** 2016-08-27

**Authors:** David A Richards, David Ekers, Dean McMillan, Rod S Taylor, Sarah Byford, Fiona C Warren, Barbara Barrett, Paul A Farrand, Simon Gilbody, Willem Kuyken, Heather O'Mahen, Ed R Watkins, Kim A Wright, Steven D Hollon, Nigel Reed, Shelley Rhodes, Emily Fletcher, Katie Finning

**Affiliations:** a Medical School, University of Exeter, Exeter, UK; b School of Psychology, University of Exeter, Exeter, UK; c Lived Experience Group, Sir Henry Wellcome Building for Mood Disorders Research, University of Exeter, Exeter, UK; d Psychological Therapy Department, Tees, Esk and Wear Valleys NHS Foundation Trust, Chester-le-Street, County Durham, UK; e Department of Health Sciences, University of York, Heslington, York, UK; f Institute of Psychiatry, Kings College London, London, UK; g Department of Psychiatry, University of Oxford, The Prince of Wales International Centre, Warneford Hospital, Oxford, UK; h Department of Psychology, Vanderbilt University, Nashville, TN, USA

## Abstract

**Background:**

Depression is a common, debilitating, and costly disorder. Many patients request psychological therapy, but the best-evidenced therapy—cognitive behavioural therapy (CBT)—is complex and costly. A simpler therapy—behavioural activation (BA)—might be as effective and cheaper than is CBT. We aimed to establish the clinical efficacy and cost-effectiveness of BA compared with CBT for adults with depression.

**Methods:**

In this randomised, controlled, non-inferiority trial, we recruited adults aged 18 years or older meeting Diagnostic and Statistical Manual of Mental Disorders IV criteria for major depressive disorder from primary care and psychological therapy services in Devon, Durham, and Leeds (UK). We excluded people who were receiving psychological therapy, were alcohol or drug dependent, were acutely suicidal or had attempted suicide in the previous 2 months, or were cognitively impaired, or who had bipolar disorder or psychosis or psychotic symptoms. We randomly assigned participants (1:1) remotely using computer-generated allocation (minimisation used; stratified by depression severity [Patient Health Questionnaire 9 (PHQ-9) score of <19 *vs* ≥19], antidepressant use, and recruitment site) to BA from junior mental health workers or CBT from psychological therapists. Randomisation done at the Peninsula Clinical Trials Unit was concealed from investigators. Treatment was given open label, but outcome assessors were masked. The primary outcome was depression symptoms according to the PHQ-9 at 12 months. We analysed all those who were randomly allocated and had complete data (modified intention to treat [mITT]) and also all those who were randomly allocated, had complete data, and received at least eight treatment sessions (per protocol [PP]). We analysed safety in the mITT population. The non-inferiority margin was 1·9 PHQ-9 points. This trial is registered with the ISCRTN registry, number ISRCTN27473954.

**Findings:**

Between Sept 26, 2012, and April 3, 2014, we randomly allocated 221 (50%) participants to BA and 219 (50%) to CBT. 175 (79%) participants were assessable for the primary outcome in the mITT population in the BA group compared with 189 (86%) in the CBT group, whereas 135 (61%) were assessable in the PP population in the BA group compared with 151 (69%) in the CBT group. BA was non-inferior to CBT (mITT: CBT 8·4 PHQ-9 points [SD 7·5], BA 8·4 PHQ-9 points [7·0], mean difference 0·1 PHQ-9 points [95% CI −1·3 to 1·5], p=0·89; PP: CBT 7·9 PHQ-9 points [7·3]; BA 7·8 [6·5], mean difference 0·0 PHQ-9 points [–1·5 to 1·6], p=0·99). Two (1%) non-trial-related deaths (one [1%] multidrug toxicity in the BA group and one [1%] cancer in the CBT group) and 15 depression-related, but not treatment-related, serious adverse events (three in the BA group and 12 in the CBT group) occurred in three [2%] participants in the BA group (two [1%] patients who overdosed and one [1%] who self-harmed) and eight (4%) participants in the CBT group (seven [4%] who overdosed and one [1%] who self-harmed).

**Interpretation:**

We found that BA, a simpler psychological treatment than CBT, can be delivered by junior mental health workers with less intensive and costly training, with no lesser effect than CBT. Effective psychological therapy for depression can be delivered without the need for costly and highly trained professionals.

**Funding:**

National Institute for Health Research.

## Introduction

Clinical depression is a common and debilitating mental health disorder, being the second largest cause of global disability.^1^ Globally, the effect of depression on aggregate economic output is predicted to be US$5·36 trillion between 2011 and 2030.^2^ Reduction of these substantial costs is a key objective for low-income, middle-income, and high-income countries alike. Antidepressant medication and cognitive behavioural therapy (CBT) have the most clinical evidence. However, although antidepressant medications are cheap, their use is limited by side-effects, poor patient adherence, and discontinuation relapse risk. CBT is as effective as are antidepressants^3^ and provides long-term protection against relapse, but it is complex and its effectiveness is dependent on the skills of psychological therapists, who are expensive to train and employ. For low-income and middle-income countries in particular, the need for an extensive professional infrastructure of such therapists limits access to CBT.

**Research in context**

**Evidence before this study**

Authors of published systematic reviews, including a Cochrane review, have commented on the limitations of existing evidence for the effectiveness of behavioural activation (BA) for depression compared with cognitive behavioural therapy (CBT) and the scarcity of cost-effectiveness data, with the existing evidence insufficiently robust to establish comparability. Authors of the Cochrane review called for studies that improve the quality of evidence. Our pretrial evidence took published review findings from the UK National Institute for Health and Care Excellence (NICE), who reported no difference in treatment outcome between BA and CBT immediately after treatment (Hedges' *g* 0·139 [95% CI −0·400 to 0·122]; p=0·296) and subsequent follow-up (0·135 [−0·456 to 0·186]; p=0·409). The authors of NICE's review regarded the existing international evidence as insufficient to recommend BA for first-line treatment in clinical guidelines for depression.

**Added value of this study**

This trial addresses these research recommendations and is, to our knowledge, the only high-quality, fully powered non-inferiority and cost-effectiveness study addressing both the effects and costs of BA compared with CBT for depression. When we combine the data from our study with data from other international studies in the meta-analysis done by NICE, our data reduce the 95% CIs around the effect size for depression symptoms immediately after treatment (Hedges' *g* 0·054 [95% CI −0·214 to 0·107]; p=0·514) and at follow-up (0·059 [−0·234 to 0·115]; p=0·503) and unequivocally show both non-inferiority of BA compared with CBT and that BA is more cost-effective than is CBT against commonly applied decision maker willingness-to-pay thresholds.

**Implications of all the available evidence**

Junior mental health workers with no professional training in psychological therapies can deliver behavioural activation, a simple psychological treatment, with no lesser effect than CBT has and at less cost. Effective psychological therapy for depression can be delivered without the need for costly and highly trained professionals.

Globally, health services require effective, easily implemented, and cost-effective psychological treatments for depression that can be delivered by less specialist health workers than are needed at present to close a treatment gap that can be as much as 80–90% in some low-income countries.^4^ One potential alternative, behavioural activation (BA), is a simple psychological treatment for depression. It might be easy and quick to train junior mental health workers (MHWs) in BA who have no professional training in psychological therapies.^5^ However, this method is only appropriate if BA delivered in this way is as effective as and more cost-effective than is CBT.

Although BA compares favourably with CBT in systematic reviews,^6^, ^7^ the existing evidence is insufficiently robust to establish comparability.^8^ Authors of a Cochrane review^7^ called for more quality studies than have been done so far and the UK National Institute for Health and Care Excellence (NICE) regarded the international evidence as insufficient to recommend BA for first-line treatment in clinical guidelines,^8^ instead recommending a large non-inferiority study: “to establish whether behavioural activation is an effective alternative to CBT”.^8^ Given these recommendations, we hypothesised that BA is non-inferior to CBT for depression treatment response in adults with depression and that BA is cost-effective compared with CBT.

## Methods

### Study design and participants

In this randomised, controlled, open-label, non-inferiority trial (the Cost and Outcome of Behavioural Activation versus Cognitive Behaviour Therapy for Depression [COBRA] trial), we recruited participants from primary care and psychological therapy services in Devon, Durham, and Leeds (UK). Eligible participants were adults aged 18 years or older who met diagnostic criteria for major depressive disorder assessed by researchers using a standard clinical interview (Structured Clinical Interview for the Diagnostic and Statistical Manual of Mental Disorders, Fourth Edition [SCID]^9^). We excluded people at interview who were receiving psychological therapy, were alcohol or drug dependent, were acutely suicidal or had attempted suicide in the previous 2 months, or were cognitively impaired, or who had bipolar disorder or psychosis or psychotic symptoms.

We recruited participants by searching the electronic case records of general practices and psychological therapy services for patients with depression, identifying potential participants from depression classification codes. Practices or services contacted patients to seek permission for researcher contact. The research team interviewed those that responded, provided detailed information on the study, took informed written consent, and assessed people for eligibility. The UK South West Research Ethics Committee gave national approval for the study (NRES/07/H1208/60). The protocol has been published previously.^10^

### Randomisation and masking

After eligibility was established, consent agreed, and baseline data collected, we randomly allocated participants (1:1) to BA or CBT using computer-generated allocation, stratified by depression severity according to the Patient Health Questionnaire 9 (PHQ-9)^11^ (<19 *vs* ≥19), antidepressant use (taking antidepressants or not), and recruitment site (Devon, Durham, or Leeds). A computer-based system allocated the first 20 participants to each group on a truly random basis. For subsequent participants, allocation was minimised to maximise the likelihood of balance in stratification variables across the two study groups. The registered Peninsula Clinical Trials Unit (Plymouth University, Plymouth, UK) allocated participants remotely after baseline data entry to ensure allocation concealment. Treatment was given open label, but outcome assessors were masked to participants' allocations. Concealment was ensured by use of an externally administered password-protected trial website with retention of a stochastic element to the minimisation algorithm. We recorded instances when outcome assessors were unmasked during interviews if participants informed them of their allocation.

### Procedures

We developed our clinical protocols in line with published treatment protocols,^12^, ^13^ including those from our own trials,^14^, ^15^ advice from international collaborators, and NICE recommendations^8^ for duration and frequency of BA and CBT. Full-time National Health Service (NHS) MHWs and therapists worked half of their working week for COBRA (with the other half worked as normal) and followed written manuals to deliver a maximum of 20 sessions over 16 weeks, with the option of four additional booster sessions if the patients wanted them.^8^ Treatment included core and supplementary techniques appropriate to the BA or CBT protocol to be used as clinically indicated; for example, behavioural or cognitive strategies for management of anxiety. All core components of both treatments were delivered by session eight, which we considered to represent a minimally sufficient dose of therapy (appendix). Sessions were face to face, lasting for 60 min. BA and CBT experts on the trial team trained MHWs and therapists for 5 days in either BA or CBT. MHWs and therapists were assessed for competence at the end of training with use of standardised quality criteria instruments consistent with the relevant treatment: either the Quality of Behavioral Activation Scale (Dimidjian S, University of Colorado, personal communication) or the Revised Cognitive Therapy Scale for CBT.^16^ Further training was given if competency was not demonstrated. MHWs and therapists received 60 min of clinical supervision fortnightly from NHS psychological therapists clinically experienced in BA or CBT, overseen by trial team experts.

Junior MHWs—graduates trained to deliver guided self-help interventions, but with neither professional mental health qualifications nor formal training in psychological therapies—delivered an individually tailored programme re-engaging participants with positive environmental stimuli and developing depression management strategies. Participants were encouraged to increase their contact with individually specified positive situations and reduce their avoidance of other situations. Specific BA techniques included identification of depressed behaviours, analysis of the triggers and consequences of depressed behaviours, monitoring of activities, development of alternative goal-orientated behaviours, scheduling of activities, and development of alternative behavioural responses to rumination.

Professional or equivalently qualified psychotherapists, accredited as CBT therapists with the British Association of Behavioural and Cognitive Psychotherapy, with a postgraduate diploma in CBT, delivered a personalised treatment programme based on an assessment of how participants' beliefs lead to emotional distress and ineffectual coping. Participants used cognitive and behavioural exercises to specifically test the accuracy of those beliefs by identifying and modifying negative thoughts and beliefs that give rise to them. Specific techniques included participants monitoring moods and activities, planning of exercises to test negative beliefs, and thought records to identify and examine the accuracy of negative automatic thoughts and underlying beliefs. We did follow-up assessments 6 months, 12 months, and 18 months after randomisation.

We assessed the quality of and adherence to treatment using audiotapes and written records of therapy sessions. Independent experts in both treatments rated a random (with use of a computer-generated random number sequence) sample of tapes, stratified by therapist, therapy session, and intervention, for competence using the Revised Cognitive Therapy Scale^16^ for CBT (range 0–72) and the Quality of Behavioral Activation Scale (Dimidjian S, University of Colorado, personal communication) for BA (range 0–96). All therapists recorded the specific therapeutic techniques that they had used for each session on a checklist.

### Outcomes

The primary outcome was self-reported depression severity (PHQ-9 score^11^) at 12 months. Secondary outcomes were PHQ-9 score at 6 months and 18 months and Diagnostic and Statistical Manual of Mental Disorders IV major depressive and anxiety disorder status and number of depression-free days between follow-ups (SCID),^9^ anxiety (Generalized Anxiety Disorder 7),^17^ and health-related quality of life (36-Item Short Form Survey)^18^ at 6 months, 12 months, and 18 months. For adverse events, we recorded deaths from whatever cause and all self-harm and suicide attempts. The independent Data Management Committee reviewed all adverse events and made relevant trial conduct recommendations.

### Statistical analysis

Previous research has suggested that non-inferiority margins should be half of the mean controlled effect size from historical trials.^19^ Accordingly, we estimated the non-inferiority margin for the primary outcome using meta-analysis data from trials of BA^14^ for which BA was superior to controls by a mean of 0·7 SD units (95% CI 0·39–1) or 3·8 PHQ-9 score units (2·1–5·4). Therefore, our non-inferiority margin was 1·9 PHQ-9 points (ie, 0·5 × 3·8). We inflated our sample size by 20% for participant follow-up attrition. We planned to recruit 220 participants per arm to detect a between-group non-inferiority margin of 1·9 PHQ-9 points with a one-sided 2·5% α. Furthermore, although findings from a trial^20^ of CBT have shown little effect of outcome clustering by therapists, the presence of a small therapist clustering effect (ie, an intracluster correlation coefficient of 0·01) would still provide the same power.

We did all analyses using a statistical analysis plan prepared in the first 6 months of the trial, agreed with the Trial Management Group, Trial Steering Committee, and Data Management Committee. We assessed between-group equivalence of baseline characteristics and outcomes descriptively and did a descriptive analysis of baseline characteristics by recruitment method.

We compared observed primary and secondary outcomes between groups 12 months after randomisation using linear regression models adjusted for baseline outcome values and stratification variables. We did modified intention-to-treat (mITT) and per-protocol (PP) analyses, as security of inference depends on both PP and intention-to-treat analyses showing non-inferiority.^21^ PP analysis provides some protection for any theoretical increase in the risk of type I error (erroneously concluding non-inferiority). Our mITT population comprises all patients according to and included in random allocation with complete data. We defined the PP population as participants meeting the mITT definition and receiving at least eight treatment sessions (representing a minimally sufficient dose of therapy). We analysed safety in the mITT population. We did sensitivity analyses for our primary outcome and for different definitions of PP (eight, 12, 16, and 20 treatment sessions) to check security of inference of non-inferiority.

We accepted non-inferiority of BA to CBT (in a 0·025 level test) if the lower bound of the two-sided 95% CI (equivalent to the upper bound of one-sided 97·5% CI) was within the non-inferiority margin of −1·9 PHQ-9 points. We checked for non-equivalence of the primary outcome at all follow-up points using the same approach.

We did secondary analyses to compare groups at follow-up across 6 months, 12 months, and 18 months using hierarchical linear regression. To ease clinical interpretation, we calculated proportions of recovery (participants with PHQ-9 scores of ≤9) and response (50% reduction from baseline PHQ-9 scores). We ran sensitivity analyses to assess the likely effect of missing data using multiple imputation models. We did imputation by treatment group using chained equations to create 20 complete datasets under the assumption that data were missing at random.^22^ Imputation models included covariates as defined for the primary analysis model and auxiliary variables that were predictive of outcomes. After analysis, we combined the effect estimates from the imputed datasets using Rubin's rule.^23^ For economic analyses, we took the UK NHS and personal social services perspective consistent with the NICE reference case,^24^ also examining a wide societal perspective, adding productivity losses due to time off work in a sensitivity analysis. We collected participants' use of BA and CBT from clinical records, with additional resource information (eg, training, supervision, and other non-face-to-face activities) from therapists and trainers. We used the Adult Service Use Schedule to measure other health and social care services used, including psychotropic medications. We measured productivity losses using the absenteeism and presenteeism questions from the Health and Work Performance Questionnaire.^25^ We calculated effectiveness in terms of quality-adjusted life-years (QALYs) using the EuroQol-5D-3L measure of health-related quality of life.^26^ We assigned health states from the EuroQol-5D-3L measure a utility score using responses from a representative sample of adults in the UK.^27^ We calculated QALYs as the area under the curve defined by the utility values at baseline and each follow-up, assuming that utility score changes over time followed a linear path.

We compared the costs and cost-effectiveness of treatments at 18 months to capture the economic effect of events like relapse with unit costs from the 2013–14 financial year.^28^, ^29^ We discounted costs and QALYs in year 2 at 3·5%.^24^ We used complete case analysis with missing data explored in a sensitivity analysis using multiple imputation with chained equations. We calculated the cost of each treatment using a microcosting (bottom-up) approach.^30^ We based MHW costs on NHS Agenda for Change salary band five (salary range £21 909–28 462; US$31 662–41 130; €27 726–35 993) for BA and band seven (£31 383–41 373; US$45 350–59 786; €39 738–52 388) for CBT therapists and included employer National Insurance and pension contributions plus capital, administrative, and managerial costs. We calculated cost per h using standard working time assumptions,^31^ weighted to account for time spent on non-patient-facing activities. We applied nationally applicable unit costs for other health and social care services.

We assessed cost-effectiveness in terms of QALYs using the net benefit approach.^32^ We analysed differences in mean cost per participant at 18 months using parametric *t* tests, with the validity of results confirmed using bias-corrected, non-parametric bootstrapping.^33^ We calculated incremental cost-effectiveness ratios and constructed cost-effectiveness planes using 1000 bootstrapped resamples from regression models of total cost and outcome by treatment group. We used these bootstrapped replications to calculate the probability that each of the treatments is the optimal choice for different values a decision maker is willing to pay for a unit improvement in outcome, representing uncertainty around the cost and effectiveness estimates, with cost-effectiveness acceptability curves illustrating the probability that BA is cost-effective compared with CBT, dependent on willingness to pay per QALY.^34^ We controlled for stratification variables and baseline values of the variables of interest, truncating data to exclude influential outliers—ie, cases with total costs in the 99th percentile that make a significant difference to the results. We did all analyses using Stata version 14.1. This trial is registered with the ISCRTN registry, number ISRCTN27473954.

### Role of the funding source

The funder of the study had no role in study design, data collection, data analysis, data interpretation, or writing of the report. DAR, RST, FCW, SB, SR, and BB had full access to all the data in the study and DAR had final responsibility for the decision to submit for publication.

## Results

**Figure 1:**
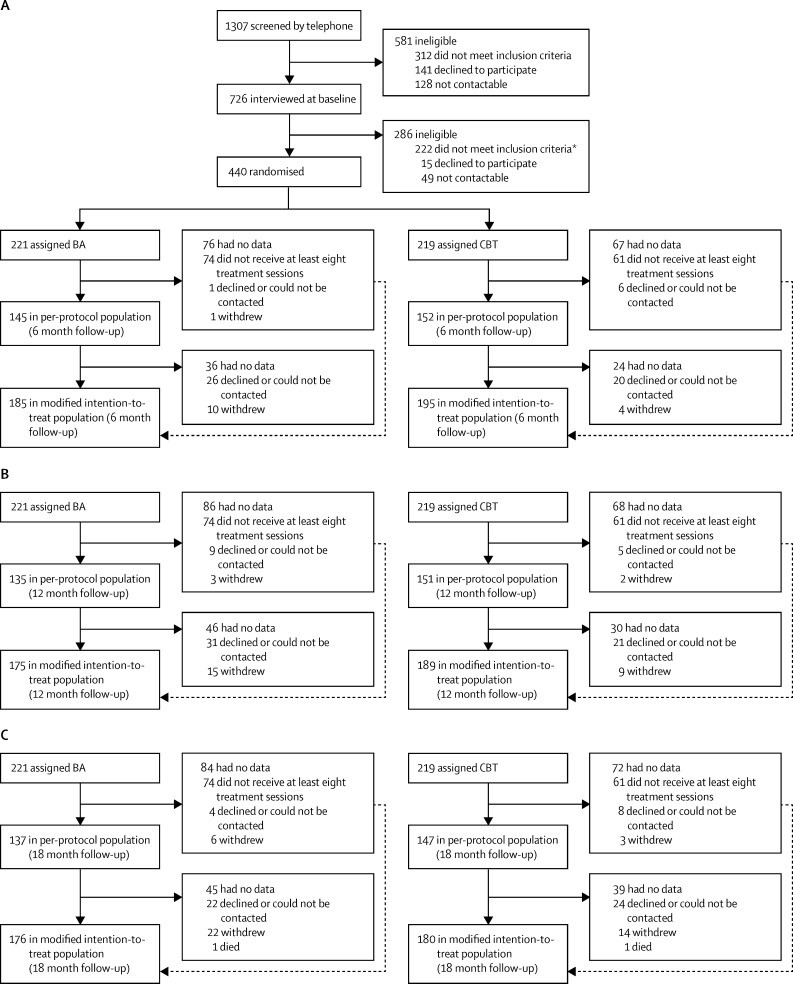
Trial profile (A) 6 month, (B) 12 month, and (C) 18 month follow-up. BA=behavioural activation. CBT=cognitive behavioural therapy. *Includes four participants who were initially allocated in error and subsequently excluded.

Between Sept 26, 2012, and April 3, 2014, we recruited 440 participants, randomly allocating 221 (50%) to the BA group and 219 (50%) to the CBT group (figure 1). 175 (79%) participants were assessable for the primary outcome in the mITT population in the BA group compared with 189 (86%) in the CBT group, whereas 135 (61%) were assessable in the PP population in the BA group compared with 151 (69%) in the CBT group. We noted no evidence of a difference in patient characteristics between recruitment methods (appendix). Patient-level and trial-level characteristics at baseline were well balanced between groups (table 1). PHQ-9 score at baseline was negatively skewed, with a high proportion of participants scoring towards the upper end of the distribution (data not shown), but scores were similar between groups (table 2).

Ten MHWs provided BA (median 22 participants each [IQR 19–25]) and 12 therapists provided CBT (21 [13–23]). MHWs had a mean of 18 months mental health experience (SD 11) and CBT therapists had a mean of 22 months post-CBT qualification (24). We removed one CBT therapist from the trial in the early stages who did not meet acceptable competency. Participants received a mean of 11·5 BA sessions (7·8) or 12·5 CBT sessions (7·8). 305 participants (69%) completed the PP number of at least eight sessions (BA 147 [67%] patients, mean 16·1 sessions [SD 5·3]; CBT 158 [72%] patients, mean 16·4 sessions [5·4]); participants completing less than eight sessions (135 [31%]; BA 74 [33%], CBT 61 [28%]) completed a mean of 2·5 BA sessions (SD 1·9) or 2·6 CBT sessions (2·1). MHWs and therapists met acceptable competency standards: mean Quality of Behavioral Activation Scale BA competence was 55 (7·5) and mean Revised Cognitive Therapy Scale for CBT competence was 37·9 (10·9).

We found no evidence of inferiority of PHQ-9 score at 12 months in either the mITT (CBT 8·4 PHQ-9 points [SD 7·5]; BA 8·4 PHQ-9 points [7·0]; mean difference 0·1 PHQ-9 points [95% CI −1·3 to 1·5]; p=0·89) or PP (CBT 7·9 PHQ-9 points [7·3]; BA 7·8 [6·5]; mean difference 0·0 [–1·5 to 1·6]; p=0·99) populations (table 2). The non-inferiority of BA to CBT was accepted for both the mITT and PP populations as the lower bound of the 95% CI (one-sided 97·5% CI) of the between-group mean difference lies within the non-inferiority margin of −1·9 PHQ-9 points (appendix). Although we initially planned to include therapist as a random-effects variable, given the low levels of observed clustering, we parsimoniously fitted our models without therapist as a variable. We checked for no inference difference with and without inclusion of a random-effects therapist term. We ruled out superiority of CBT to BA as the lower bound of the 95% CI included zero for the mITT and PP populations. The inference of non-inferiority was robust to sensitivity analysis across different PP definitions. We found no evidence of a significant between-group treatment interaction across the mITT or PP populations with the primary outcome at 12 months as stratified by depression severity, antidepressants use, and recruitment site (appendix).

We found that BA was not different from CBT in anxiety (Generalized Anxiety Disorder 7), depression status, and depression-free days and anxiety diagnoses (SCID) for either the mITT or PP populations using observed or imputed data at 12 months (table 2). Because of substantial missing 36-Item Short Form Survey data at baseline, we analysed these data adjusted for stratification variables only. We found no difference in numbers of participants with at least one anxiety diagnosis: BA 43 (28%) of 153; CBT 43 (27%) of 161 (mITT population; χ^2^ 0·08; p=0·78).

Between 61% and 70% of mITT and PP participants in both groups met criteria for recovery from depression or response to treatment at 12 months, with no differences in the proportions of patients in each group who recovered or responded (table 3). Using observed data for all outcomes, we found no evidence of a difference between the CBT and BA groups over the period of the trial, as indicated by a non-significant time-by-treatment effect interaction, for both the mITT and PP populations (appendix). We found a small, negligible clustering of primary and secondary outcome scores at follow-up across therapists overall and within BA and CBT groups (intracluster correlation coefficient ≤0·04).

Two (1%) non-trial-related deaths (one [1%] multidrug toxicity in the BA group and one [1%] cancer in the CBT group) and 15 depression-related, but not treatment-related, serious adverse events (three in the BA group and 12 in the CBT group) occurred in three [2%] participants in the BA group (two [1%] patients who overdosed and one [1%] who self-harmed) and eight (4%) participants in the CBT group (seven [4%] who overdosed and one [1%] who self-harmed). Of the 440 participants recruited, 76 (17%) had missing primary outcome data at 12 month follow-up. The proportion of missing PHQ-9 data was higher in the BA than in the CBT group (46 [21%] *vs* 30 [14%]; odds ratio 1·6 [95% CI 1·0–2·7]; p=0·05). Imputation of data for primary and secondary outcomes at 12 months showed that in accordance with the observed data analysis, no difference existed between groups (Table 2, Table 3), supporting our conclusion of non-inferiority. The odds of missing PHQ-9 data were higher for patients with increased baseline severity of depression (PHQ ≥19, odds ratio 1·6 [95% CI 1·0–2·6]; p=0·05) and increasing age (in years) was associated with lower odds of missing PHQ-9 data (odds ratio 0·97 [0·96–0·99]; p=0·01). We found no evidence of an association between missingness and any other baseline characteristic (data not shown). Outcome assessors reported having been unmasked for 16 (4%) participants (five [2%] in the BA group and 11 [5%] in the CBT group; due to participants informing assessors of their treatment allocation).

**Figure 2:**
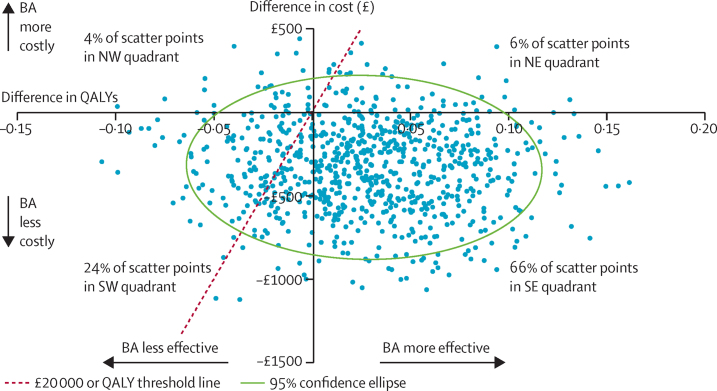
Bootstrapped mean differences in costs and effects of BA compared with CBT BA=behavioural activation. CBT=cognitive behavioural therapy. NE=northeast. NW=northwest. SE=southeast. SW=southwest. QALY=quality-adjusted life-year.

For economic analyses, at 18 months, full service use data was available for 159 (90%) of 176 participants in the BA group and 168 (93%) of 180 participants in the CBT group. We found a significant difference in mean intervention costs between the two groups, but no differences in other categories of cost or in total cost (table 4). Mean health state utility scores according to EuroQoL-5D-3L were slightly higher in the BA group than in the CBT group across the entire follow-up period, with resultant QALYs also higher for BA, but the QALY difference was not significant. Costs were lower and QALY outcomes better in the BA group than in the CBT group, generating an incremental cost-effectiveness ratio of –£6865. The scatterplot of bootstrapped cost and effectiveness pairs for BA versus CBT illustrates dominance of BA over CBT, with the point estimate and two-thirds of scatter points falling in the southeast quadrant of the cost-effectiveness plane, where BA replications are cheaper and more effective than are CBT ones (figure 2). The cost-effectiveness acceptability curve (appendix) showing the probability of BA being cost-effective compared with CBT does not fall below 75% and is closer to 80% at NICE-preferred willingness^24^ to pay £20 000–30 000 per QALY.

In all sensitivity analyses, including complementary therapies and productivity losses, as well as analyses taking narrow intervention and mental health service perspectives, BA was significantly less costly than was CBT, so BA continues to have a higher probability of being cost-effective than does CBT at the NICE threshold (appendix).^24^ Imputation of missing data increased the difference in total cost (BA £1841·67; CBT £2282·40; difference –£440·73 [95% CI −1007·71 to 126·26]; p=0·13), but reduced the difference in QALYs (BA 1·22; CBT 1·19; difference 0·03 [–0·06 to 0·11]; p=0·55), increasing the incremental cost-effectiveness ratio to –£16 951. The cost-effectiveness acceptability curve for the missing data analysis again supported the likelihood that BA is cost-effective compared with CBT (appendix).

## Discussion

We found that BA for depression is not inferior to CBT in terms of reduction of depression symptoms and is more cost-effective than is CBT against commonly applied decision maker willingness to pay thresholds. We observed our results using both mITT and PP analyses, using a conservative non-inferiority margin. Our economic analyses were driven by the lower costs of the MHWs who delivered BA compared with the more experienced psychological therapists who routinely deliver CBT. Our study results therefore substantiate the hypothesis that BA is as effective as is CBT and that its simplicity renders BA suitable for delivery by junior MHWs with no professional training in psychological therapies.^5^

This trial is the largest trial of BA to date and is one of the largest trials of psychological treatments for depression. We followed up participants for 18 months and our economic analysis is one of few in this field. Therapists and MHWs working in three different routine UK care settings delivered treatment, providing evidence of potential generalisability. We assessed therapy quality using independent raters and ensured that treatment in both arms was delivered to the standard recommended guidelines. Our levels of attrition and outcome loss to follow-up were low at 12 months and 18 months, similar to other trials in this area, but are still a limitation. Although participants in the per-protocol population attended similar numbers of sessions to those in other CBT trials,^15^ 35% of participants chose to not even attend a minimal number of sessions, a problem well known to routine psychological therapy services. This pragmatic trial done in routine environments means that we were unable to quantify or control for the contribution of antidepressant medicines to outcomes. However, most participants who were taking medication had been doing so for a considerable time before entering the trial, making it unlikely that our results were driven by pharmacological treatment. Given the nature of the intervention and comparator, we could not mask patients or the mental health workers or therapists who were delivering the interventions to treatment allocation, but we used self-reported outcome measures and robust outcome assessor-masking procedures to reduce researcher unmasking to less than 5%. Missing data for the primary outcome measure was substantial. However, our between-group inferences were robust to data imputation.

Our findings could have substantial implications for the scalability of psychological treatment for depression internationally^4^ given the greater availability and ease with which a BA workforce could be trained than could a CBT workforce. For many years, CBT has been the foremost psychological therapy recommended by therapists, researchers, and policy makers. Our results challenge this dominance. Although more work needs to be done than has been done so far to find ways to effectively treat the 20–23% of patients whose depression was unchanged by BA or CBT, our findings suggest that BA should be a front-line treatment for depression, with substantial potential to improve reach and access to psychological therapy globally.

Our results in both groups compare favourably with a meta-analysis^3^ of the effects of CBT that estimate proportions of patients with remissions of around 50%. Our cost-effectiveness analyses show the high probability that BA is cost-effective and affordable compared with CBT at standard willingness to pay thresholds. Our most striking finding is that BA leads to similar clinical outcomes for patients with depression, but at a financial saving to clinical providers of 21% compared with the costs of provision of CBT, with no compensatory use of other health-care services by patients.

Driving these savings is the fact that BA can be delivered by inexperienced MHWs with no professional training in psychological therapies, with no lesser effect than that of more highly trained and experienced psychological therapists giving patients CBT. Although many obstacles exist to successful dissemination in addition to training of MHWs, our findings suggest that health services globally could reduce the need for costly professional training and infrastructure, reduce waiting times, and increase access to psychological therapies.^4^ Our findings have substantial implications given the increasing global pressure for cost containment across health systems in high-income countries and the need to develop accessible, scalable interventions in low-income and middle-income countries. Such countries might choose to investigate the training and employment of junior workers over expensive groups of psychological professionals. Our results, therefore, offer hope to many societies, cultures, and communities worldwide, rich and poor, struggling with the effect of depression on the health of their people and economies.

## Figures and Tables

**Table 1:** Baseline characteristics

		**BA (n=221)**	**CBT (n= 219)**	**All (n=440)**
**Trial characteristics**				
Method of recruitment				
	Primary care	192 (87%)	190 (87%)	382 (87%)
	IAPT	29 (13%)	29 (13%)	58 (13%)
**Patient characteristics**				
Age (years)		43.9 (14·1)	43·0 (14·1)	43·5 (14·1)
Sex				
	Male	79 (36%)	71 (32%)	150 (34%)
	Female	142 (64%)	148 (68%)	290 (66%)
Number of episodes of depression (including current)				
	Mean	7·0 (15·0)	6·3 (13·8)	6·7 (14·4)
	Median	3·0 (1–5)	2·0 (1–5)	3·0 (1–5)
Age of onset of first depression episode (years)		27·2 (15·0)	26·3 (13·5)	26·7 (14·2)
Duration of antidepressant treatment (weeks)*				
	Mean; n	215 (817); 160	116 (480); 169	164 (666); 329
	Median; n	21 (10–78); 160	18 (7–52); 169	19 (8–71); 329
At least one comorbid anxiety disorder		131 (59%)	141 (64%)	272 (62%)
Marital status				
	Single	68 (31%)	59 (27%)	127 (29%)
	Cohabiting (not married)	29 (13%)	25 (11%)	54 (12%)
	Civil partnership	1 (<1%)	1 (<1%)	2 (<1%)
	Married	84 (38%)	92 (42%)	176 (40%)
	Divorced or separated	39 (18%)	42 (19%)	81 (18%)
Number of children				
	0	74 (33%)	72 (33%)	146 (33%)
	1	35 (16%)	31 (14%)	66 (15%)
	2	67 (30%)	69 (32%)	136 (31%)
	3	31 (14%)	27 (12%)	58 (13%)
	≥4	14 (6%)	20 (9%)	34 (8%)
Level of education				
	No qualifications	25 (11%)	30 (14%)	55 (13%)
	GCSEs or O Levels	36 (16%)	43 (20%)	79 (18%)
	AS or A Levels	28 (13%)	22 (10%)	50 (11%)
	NVQ or other vocational qualification	54 (24%)	71 (32%)	125 (28%)
	Undergraduate degree	44 (20%)	35 (16%)	79 (18%)
	Postgraduate degree	28 (13%)	14 (6%)	42 (10%)
	Doctoral degree	2 (1%)	1 (<1%)	3 (1%)
	Professional degree (eg, MD)	4 (2%)	3 (1%)	7 (2%)
Ethnicity				
	White British	204 (92%)	197 (90%)	402 (91%)
	Other	17 (8%)	22 (10%)	38 (9%)
**Stratification or minimisation variables**				
PHQ-9 category				
	<19	118 (53%)	118 (54%)	236 (54%)
	≥19	103 (47%)	101 (46%)	204 (46%)
Antidepressant use				
	Yes	172 (78%)	173 (79%)	345 (78%)
	No	49 (22%)	46 (21%)	95 (22%)
Site				
	Devon	74 (33%)	73 (33%)	147 (33%)
	Durham	79 (36%)	78 (36%)	157 (36%)
	Leeds	68 (31%)	68 (31%)	136 (31%)

Data are n (%), mean (SD), or median (IQR), unless otherwise indicated. IAPT=Improving Access to Psychological Therapies. GCSE=General Certificate of Secondary Education. O Level=Ordinary Level. AS Level=Advanced Subsidiary Level. A Level=Advanced Level. NVQ=National Vocational Qualification. MD=Doctor of Medicine. PHQ-9=Patient Health Questionnaire 9.

* 16 participants who reported that they were using antidepressant medication at baseline did not report duration of use (12 in the BA group and four in the CBT group).

**Table 2:** Primary and secondary outcomes at 12 months

		**CBT**	**BA**	**Observed data only**		**Observed and imputed data**	
				Between-group difference	p value	Between-group difference	p value
**Primary outcome**							
PHQ-9							
	Baseline	17·4 (4·8); 219	17·7 (4·8); 221	..		..	
	mITT	8·4 (7·5); 189	8·4 (7·0); 175	0·1 (−1·3 to 1·5)*	0·89	0·2 (−1·1 to 1·7)*	0·80
	PP	7·9 (7·3); 151	7·8 (6·5); 135	0·0 (−1·5 to 1·6)*	0·99	0·0 (−1·6 to 1·6)*	0·99
**Secondary outcomes**							
GAD-7							
	Baseline	12·6 (5·1); 219	12·7 (5·1); 221	..		..	
	mITT	6·3 (6·0); 176	6·4 (5·9); 161	−0·1 (−1·0 to 1·3)*	0·82	0·0 (−1·3 to 1·4)*	0·96
	PP	6·0 (5·8); 146	5·9 (5·5); 129	0·01 (−1·3 to 1·2)*	0·95	−0·4 (−1·7 to 1·0)*	0·60
SCID number of depression-free days							
	Baseline	..	..	..		..	
	mITT	129 (58); 160	120 (56); 150	9 (−3 to 23)*	0·13	7 (−7 to 20)*	0·27
	PP	132 (55); 138	119 (55); 125	13 (0 to 26)*	0·06	8 (−4 to 21)*	0·21
SF-36v2 PCS							
	Baseline	50·1 (13·1); 65	51·4 (11·9); 69	..		..	
	mITT	48·1 (12·2); 168	49·9 (11·6); 150	1·6 (−1·0 to 4·2)†	0·22	1·4 (−1·1 to 4·0)†	0·27
	PP	48·0 (12·2); 144	49·9 (12·0); 125	1·6 (−1·3 to 4·4)†	0·28	1·3 (−1·5 to 4·1)†	0·36
SF-36v2 MCS							
	Baseline	23·2 (9·4); 65	22·5 (7·8); 69	..		..	
	mITT	41·7 (14·1); 168	41·6 (14·0); 150	0·0 (−3·0 to 3·0)†	0·99	0·0 (−2·9 to 2·8)†	0·97
	PP	42·9 (13·6); 144	42·3 (13·3); 125	−0·5 (−3·7 to 2·7)†	0·77	−0·6 (−3·8 to 2·7)†	0·73

Data are mean (SD); n or mean (95% CI). CBT=cognitive behavioural therapy. BA=behavioural activation. PHQ-9=Patient Health Questionnaire 9. mITT=modified intention to treat. PP=per protocol. GAD-7=Generalized Anxiety Disorder 7. SCID=Structured Clinical Interview for the Diagnostic and Statistical Manual of Mental Disorders, Fourth Edition. SF-36v2=36-Item Short Form Survey version 2. PCS=physical component summary. MCS=mental component summary.

* Models adjusted for baseline outcome score and stratification variables (symptom severity [PHQ-9 score of <19 *vs* ≥19], site [Devon, Durham, or Leeds], and antidepressant use [use or not]).

† Models adjusted for stratification variables, but not baseline outcome score because of substantial missing data.

**Table 3:** Depression status, recovery, and response at 12 months

	**CBT**	**BA**	**Observed data only**		**Observed and imputed data**	
			Odds ratio	p value	Odds ratio	p value
**SCID depression**						
Baseline	219/219 (100%)	221/221 (100%)				
mITT	37/163 (23%)	31/154 (20%)	0·9 (0·5–1·6)	0·71	0·9 (0·5–1·6)	0·70
PP	30/141 (21%)	24/128 (19%)	0·9 (0·5–1·7)	0·80	0·9 (0·5–1·7)	0·75
Depression recovery*						
mITT	124/189 (66%)	115/175 (66%)	1·0 (0·6–1·5)	0·96	1·2 (0·7–1·9)	0·53
PP	104/151 (69%)	94/135 (70%)	1·0 (0·6–1·7)	0·96	1·2 (0·7–2·0)	0·47
**Depression response**†						
mITT	117/189 (62%)	107/175 (61%)	1·0 (0·9–1·1)	0·73	0·9 (0·6–1·4)	0·75
PP	100/151 (66%)	87/135 (64%)	0·9 (0·9–1·0)	0·64	0·9 (0·5–1·4)	0·55

Data are n/N (%) or odds ratio (95% CI). CBT=cognitive behavioural therapy. BA=behavioural activation. SCID=Structured Clinical Interview for the Diagnostic and Statistical Manual of Mental Disorders, Fourth Edition. mITT=modified intention to treat. PP=per protocol.

* Participants with Patient Health Questionnaire 9 scores of 9 or less.

† Participants with a 50% reduction from baseline in Patient Health Questionnaire 9 score.

**Table 4:** Economic data at 18 months

	**BA**	**CBT**	**Difference**	**p value**
**Costs per participant (£)**				
Intervention	£974·81 (475·02); 159	£1235·23 (610·03); 168	−£262·29 (−381·40 to −143·19)	<0·0001
Hospital	£860·23 (1509·88); 159	£927·26 (1975·64); 168	−£75·67 (−451·75 to 300·42)	0·69
Community health and social care	£644·36 (816·07); 159	£944·25 (1726·17); 168	−£15·14 (−304·90 to 274·62)	0·91
Medication	£103·20 (197·92); 159	£117·64 (265·92); 168	£2·15 (−39·83 to 44·13)	0·92
Total	£2596·62 (1846·72); 159	£3250·74 (3040·99); 168	−£343·24 (−857·62 to 171·13)	0·19
**EQ-5D-3L utility score**				
Baseline	0·548 (0·307); 159	0·474 (0·317); 168	..	..
6 months	0·683 (0·310); 153	0·677 (0·310); 151	..	..
12 months	0·684 (0·341); 147	0·671 (0·348); 156	..	..
18 months	0·670 (0·311); 152	0·624 (0·335); 157	..	..
QALYs	0·985 (0·422); 152	0·935 (0·433); 157	0·050 (−0·046 to 0·145)	0·31

Data are mean (SD); n or mean difference (95% CI). BA=behavioural activation. CBT=cognitive behavioural therapy. EQ=EuroQol. QALY=quality-adjusted life-year.
