# Supplementary material for: Cost and Outcome of Behavioural Activation versus Cognitive Behavioural Therapy for Depression (COBRA): a randomised, controlled, non-inferiority trial
Source: Lancet. 2016 Aug 27;388(10047):871–80. doi: 10.1016/S0140-6736(16)31140-0 (PMC5007415; doi:10.1016/S0140-6736(16)31140-0)
Supplement: Supplementary appendix [file mmc1.pdf]

THE LANCET

Supplementary appendix

This appendix formed part of the original submission and has been peer reviewed. We post it as supplied by the authors.

Supplement to: Richards DA, Ekers D, McMillan D, et al. Cost and Outcome of Behavioural Activation versus Cognitive Behavioural Therapy for Depression (COBRA): a randomised, controlled, non-inferiority trial. *Lancet* 2016; published online July 22. http://dx.doi.org/10.1016/S0140-6736(16)31140-0.

Supplementary Appendix

BA and CBT Clinical Protocol Schematics

mITT repeated measures graphs

PP repeated measures graphs

Baseline trial, patient, and minimisation characteristics by recruitment method

Figure: mean difference and 2-sided 95% confidence interval for the primary outcome of PHQ-9 at 12-month and non-inferiority margin

Subgroup analyses on the primary outcome at 12-months

Cost-effectiveness acceptability curve showing the probability that BA is cost-effective compared to CBT for different values of willingness to pay per QALY

Results of sensitivity analyses of economic evaluation

Cost-effectiveness acceptability curve showing the probability that BA is cost-effective compared to CBT for different values of willingness to pay for a QALY, including imputed missing data

6. Behavioural Activation Protocol Overall Session Chart

PHASE I						TRANSITION	PHASE II								TRANSITION	PHASE III				BOOSTER			
1	2	3	4	5	6	7	8	9	10	11	12	13	14	15	16	17	18	19	20	21	22	23	24
Assessment/rationale Formulation diagram																							
	Goal setting and introduction of valued activities																						
Self-monitoring leading to activity scheduling																							
			Avoidance-Functional analysis/TRAP and TRAC; developing formulation-diagram																				
						Review A What have learnt/target and hierarchy for next phase of valued activities				Mini progress review by now					Review B What have learnt/target and hierarchy for next phase of valued activities				Review What have learnt/target and hierarchy for next phase of valued activities				
						Carry on Activating (up your hierarchy….) including grading and stress testing																	
						<u>Additional module choices guided by functional analysis (**mandatory**/optional)</u><br>**Rumination;**<br>**Problem solving;**<br>Functional equivalence (including values);<br>Anxiety;<br>Punishment;<br>Communication;<br>Alcohol and Substance Use																	
																Relapse Prevention/ Maintaining Progress							

6. CBT Protocol Overall Session Chart

PHASE I						TRANSITION	PHASE II								TRANSITION	PHASE III				BOOSTER			
1	2	3	4	5	6	7	8	9	10	11	12	13	14	15	16	17	18	19	20	21	22	23	24
Assessment/rationale/ agreed presenting issues/shaping towards goals. Descriptive case formulation diagram																							
	Goal setting and first interventions					Progress to goals reviewed									Progress to goals reviewed								
Homework																							
Behavioural experiments																							
	Behavioural interventions: Activity and mastery and scheduling pleasurable /rewarding activities																						
			Identifying and responding to automatic thoughts																				
						Identifying conditional assumptions and using cognitive and behavioural strategies to reframe conditional assumptions and articulate and test out more adaptive beliefs. Cross-sectional case formulation.																	
															Longitudinal case formulation, only if necessary, identifying and working with core beliefs, again only if necessary.								
																Relapse Prevention/ Maintaining Progress							

Dark blue = core activities for these sessions; Light blue = non-core, but optional activities for these sessions

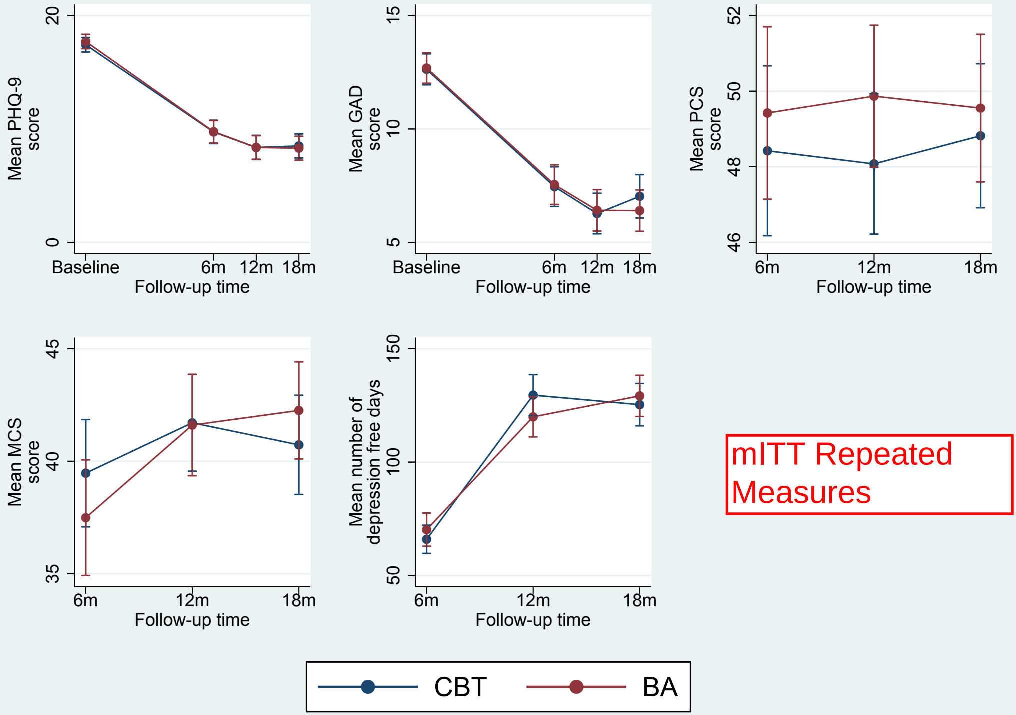

Mean PHQ-9 score
20
0
Baseline
6m
12m
18m
Follow-up time
Mean GAD score
15
10
5
Baseline
6m
12m
18m
Follow-up time
Mean PCS score
52
50
48
46
6m
12m
18m
Follow-up time
Mean MCS score
45
40
35
6m
12m
18m
Follow-up time
Mean number of depression free days
150
100
50
6m
12m
18m
Follow-up time
mITT Repeated Measures
CBT
BA

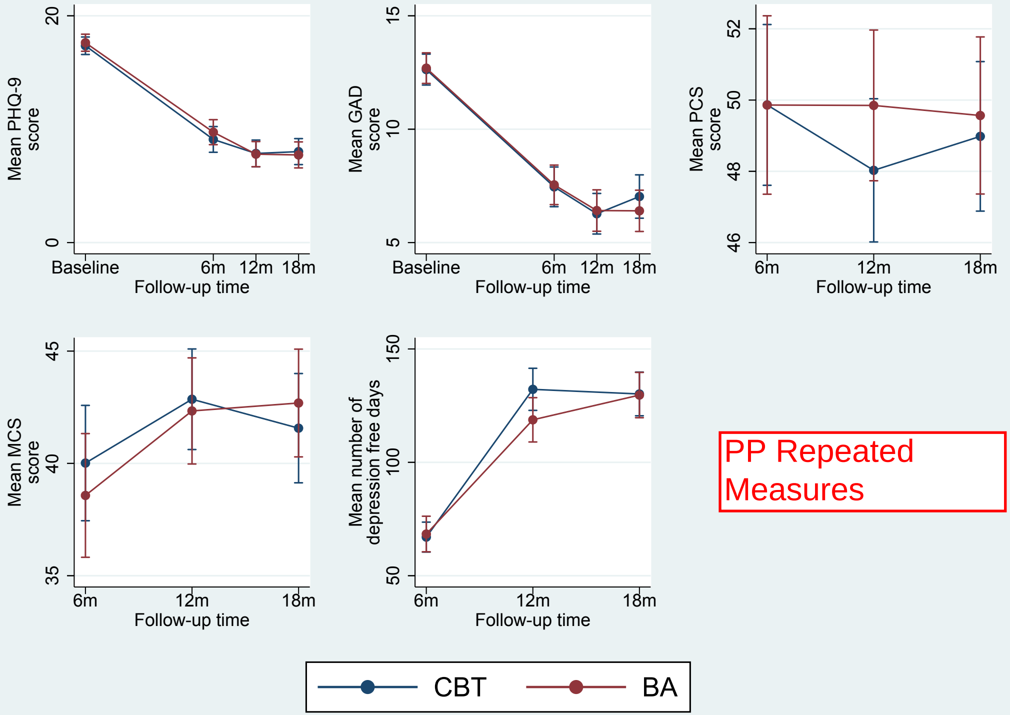

Mean PHQ-9 score
0
20
Baseline
6m
12m
18m
Follow-up time
Mean GAD score
5
10
15
Baseline
6m
12m
18m
Follow-up time
Mean PCS score
46
48
50
52
6m
12m
18m
Follow-up time
Mean MCS score
35
40
45
6m
12m
18m
Follow-up time
Mean number of depression free days
50
100
150
6m
12m
18m
Follow-up time
PP Repeated Measures
CBT
BA

Recruitment method	Primary Care (n=382)	IAPT (n= 58)	All (n=440)
**Patient characteristics**			
**Age** (years)	43·6 (14·2)	42·7 (13·4)	43·5 (14·1)
**Sex**			
Male	122 (32%)	28 (48%)	150 (34%)
Female	260 (68%)	30 (52%)	290 (66%)
**Number of episodes of depression (including current)**			
Mean	6.9 (15.3)	5·6 (7·2)	6·7 (14·4)
Median	3·0 (1-5)	3.0 (2-5)	3·0 (1-5)
**Age of onset of first depression episode (years)**	27·0 (14.6)	25·6 (13·2)	26·7 (14·4)
**Duration of antidepressant treatment (weeks)**[a]			
Mean; n	163 (697); 294	167 (313); 35	164 (666); 329
Median; n	19 (8-69); 294	23 (15-108); 35	19 (8-71); 329
**Marital Status**			
Single	112 (29%)	15 (26%)	127 (29%)
Married/Cohabiting/Civil Partnership	196 (51%)	36 (62%)	232 (53%)
Divorced/Separated	74 (19%)	7 (12%)	81 (18%)
**Number of children**			
0	123 (32%)	23 (40%)	146 (33%)
1	58 (15%)	8 (14%)	66 (15%)
2	121 (32%)	15 (26%)	136 (31%)
3	49 (13%)	9 (16%)	58 (13%)
≥4	31 (8%)	3 (5%)	34 (8%)
**Level of education**			

No qualifications	50 (13%)	5 (9%)	55 (13%)
GCSEs/O-Levels	71 (19%)	8 (14%)	79 (18%)
AS/A-Levels	44 (12%)	6 (10%)	50 (11%)
NVQ or other vocational qualification	106 (28%)	19 (33%)	125 (28%)
Undergraduate degree	66 (17%)	13 (23%)	79 (18%)
Postgraduate degree	36 (9%)	6 (10%)	42 (10%)
Doctoral degree	3 (1%)	0 (0%)	3 (1%)
Professional degree (e.g. MD)	6 (2%)	1 (2%)	7 (2%)
**Ethnicity**			
White: British	353 (92%)	49 (84%)	402 (91%)
Other	29 (76%)	9 (2%)	38 (9%)
**Stratification or minimisation variables**			
**PHQ-9 category**			
<19	200 (52%)	36 (62%)	236 (54%)
≥19	182 (48%)	22 (38%)	204 (46%)
**Antidepressant use**			
Yes	309 (81%)	36 (62%)	345 (78%)
No	73 (19%)	22 (38%)	95 (22%)
**Site**			
Devon	145 (38%)	2 (4%)	147 (33%)
Durham	157 (41%)	0 (0%)	157 (36%)
Leeds	80 (21%)	56 (97%)	136 (31%)

Data are n (%), mean (SD), or median (IQR), unless otherwise indicated. IAPT=Improving Access to Psychological Therapies. GCSE=General Certificate of Secondary Education. O Level=Ordinary Level. AS Level=Advanced Subsidiary Level. A Level=Advanced Level. NVQ=National Vocational Qualification. MD=Doctor of Medicine. PHQ-9=Patient Health Questionnaire 9.[a]16 participants who reported that they were using antidepressant medication at baseline did not report duration of use, 15 primary care and 1 IAPT.

Supplementary Table: Baseline trial, patient, and minimisation characteristics by recruitment method

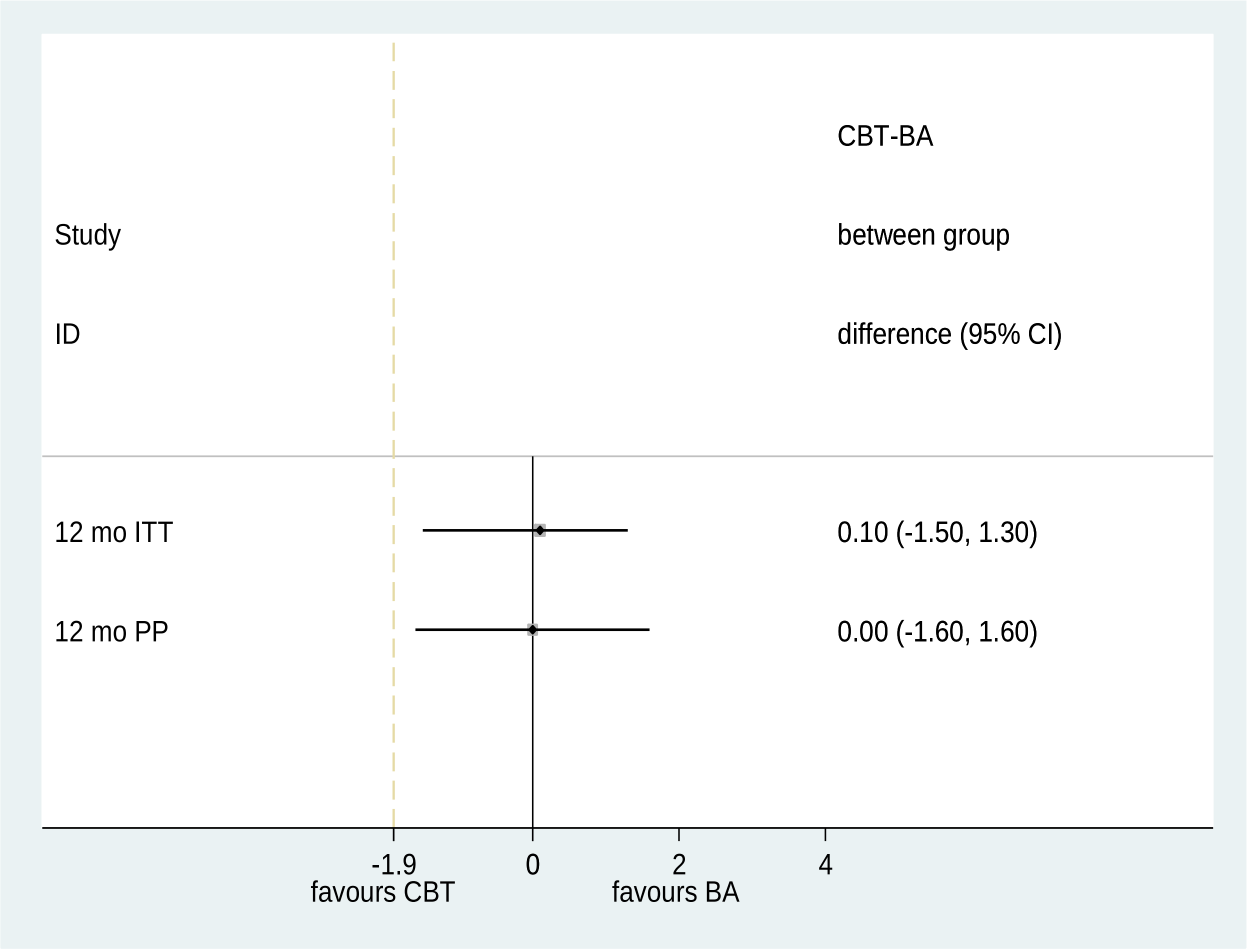

Non-inferiority of BA compared to CBT is accepted for ITT and PP analysis as the lower bound of the 95% confidence interval (one-sided 97.5% CI) lies within the non-inferiority margin of -1.90 (dotted line) in PHQ9 score. Superiority is ruled out as the lower bound of the 95% confidence interval excludes zero.

Figure 2: Mean difference and 2-sided 95% confidence interval for the primary outcome of PHQ-9 at 12-month and non-inferiority margin

	ITT population		PP population	
	**Between group difference*** Mean (95% CI)	**Interaction coefficient** (95% CI) P-value	**Between group difference*** Mean (95% CI)	**Interaction coefficient** (95% CI) P-value
**Depression severity**				
PHQ-9 < 19	0.4 (-1.3 to 2.0)		0.7 (-1.1 to 2.6)	
PHQ-9 ≥ 19	-0.6 (-3.5 to 1.8)	1.1 (-1.8 to 3.9), 0.48	-0.9 (-3.5 to 1.7)	1.9 (-1.2 to 5.0), 0.23
**Receiving anti-depressive medication**				
Yes	0.2 (-2.7 to 3.1)		-0.4 (-3.5 to 2.7)	
No	-0.1 (-1.7 to 1.5)	0.2 (-3.2 to 3.7), 0.90	0.2 (-1.6 to 2.0)	0.6 (-4.3 to 3.2), 0.74
**Site**				
1 Exeter	-1.2 (-3.6 to 1.1)		-1.2 (-3.9 to 1.5)	
2 Durham	1.0 (-1.6 to 3.6)	-2.1 (-5.5 to 1.4)	0.6 (-2.2 to 3.4)	-1.7 (-5.5 to 2.1),
3 Leeds	-0.2 (-2.8 to 2.3)	-0.9 (-4.5 to 2.6), 0.49^	0.2 (-2.3 to 3.0)	-1.3 (-5.3 to 2.6), 0.64^

*All models adjusted for baseline outcome score, and stratification variables (i.e., symptom severity (PHQ < 19, PHQ ≥ 19), site (Devon, Durham, Leeds), antidepressant use (currently taking anti-depressant medication, not currently taking anti-depression medication)

^Global P-value

Supplementary Table. Subgroup analyses on the primary outcome at 12-months

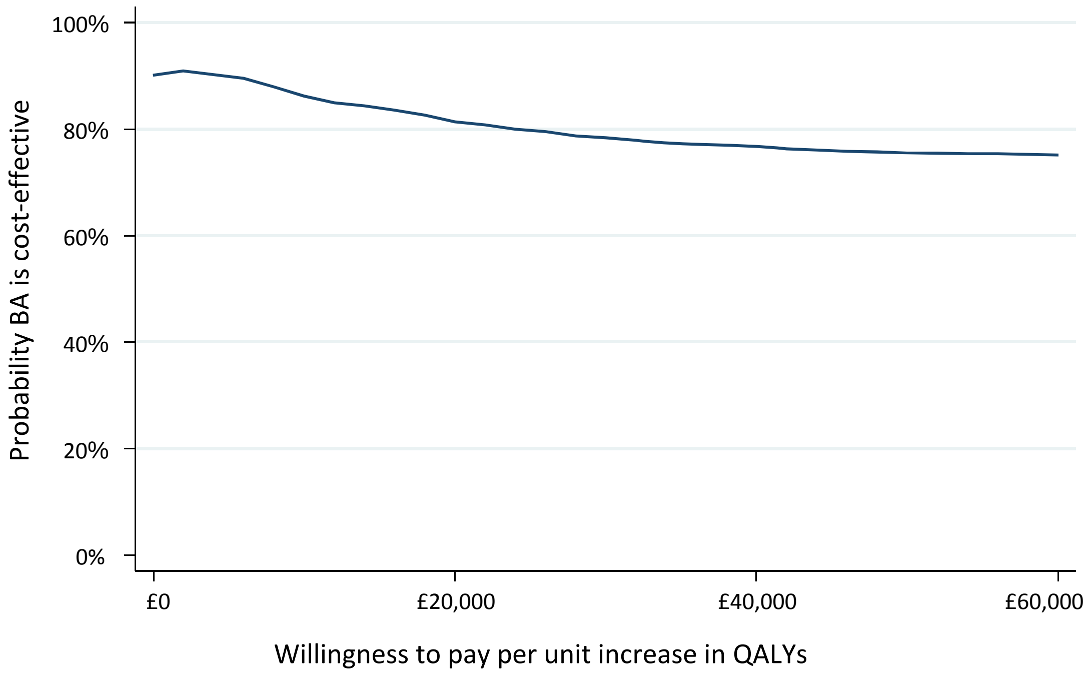

Supplementary Figure: Cost-effectiveness acceptability curve showing the probability that BA is cost-effective compared to CBT for different values of willingness to pay per QALY

	BA		CBT				
	n	Mean (SD)	n	Mean (SD)	Mean difference	95% CI	p-value
Impact on total costs (£)							
Main analysis	159	2596.62 (1846.72)	168	3250.74 (3040.99)	-343.24	-857.62 to 171.13	0.190
Inclusion of complementary therapies	158	1651.77 (259.06)	168	2187.37 (383.08)	-535.60	-1045.28 to -25.92	0.039
Inclusion of productivity losses	152	1648.43 (581.64)	166	3374.80 (837.28)	-1726.37	-2870.8 to -581.93	0.003
Intervention perspective	159	992.73 (60.54)	168	1255.03 (88.16)	-262.30	-381.40 to -143.19	<0.0001
Mental health care perspective	159	914.71 (67.86)	168	1253.85 (99.97)	-339.14	-472.64 to -205.64	<0.0001
Imputation of missing data	221	1841.67 (287.97)	219	2282.40 (423.94)	-440.73	-1007.70 to 126.26	0.127
Impact on QALYs							
Main analysis	152	0.984 (0.422)	157	0.935 (0.433)	0.050	-0.046 to 0.145	0.308
Imputation of missing data	221	1.224 (0.043)	219	1.198 (0.061)	0.026	-0.058 to 0.109	0.546

Supplementary Table. Results of sensitivity analyses of economic evaluation

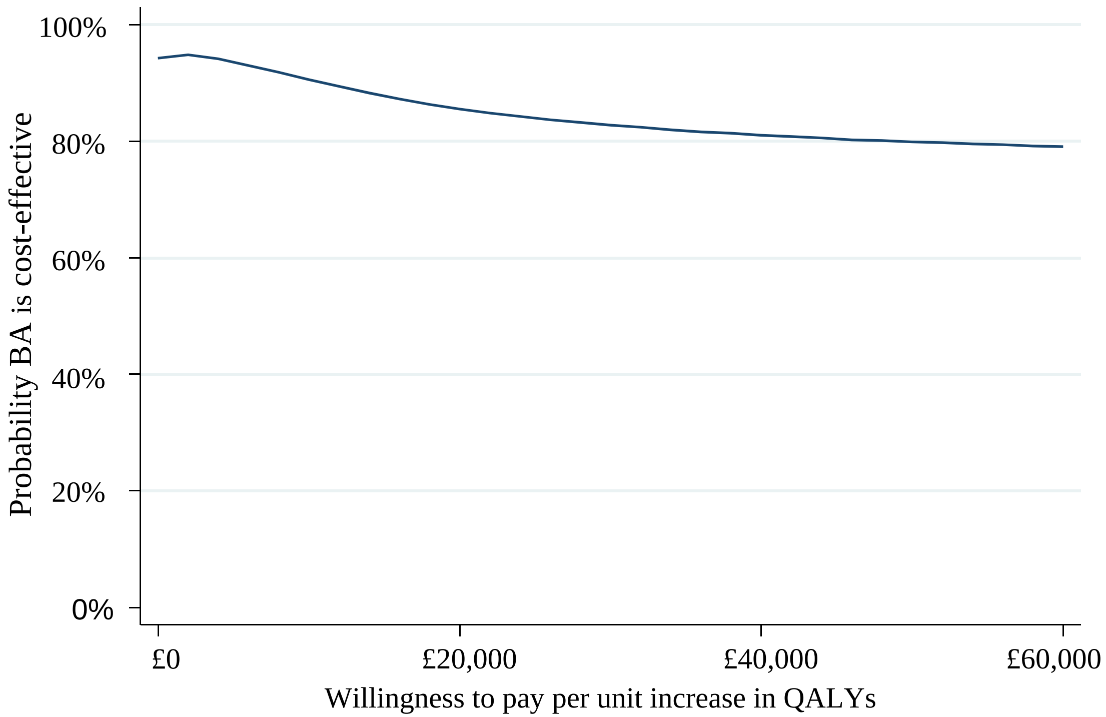

Supplementary Figure. Cost-effectiveness acceptability curve showing the probability that BA is cost-effective compared to CBT for different values of willingness to pay for a QALY, including imputed missing data
